# AGE DIFFERENCES IN DEATH ATTITUDES IN THE CONTEXT OF MULTIDIMENSIONAL TORNADO IMPACTS

**DOI:** 10.1093/geroni/igae098.2369

**Published:** 2024-12-31

**Authors:** Zhirui Chen, Zhen Cong

**Affiliations:** Boston College, Watertown, Massachusetts, United States; University of Alabama at Birmingham, Birmingham, Alabama, United States

## Abstract

This study examined the age differences in various attitudes towards death following tornado exposures, as well as the moderating effect of multidimensional tornado impacts. Data used was from the first wave of longitudinal study “Vulnerability and Resilience to Disasters” (N = 1,067). Latent class analysis was conducted to explore the underlying patterns of negative tornado impacts based on 15 indicators. Three latent classes were identified: low overall impacts with high power outage rate (Low-PO), moderate overall impacts with high emotional distress (Moderate-ED), and severe overall impacts. Subsequently, the results of linear regressions showed that relative to young-old adults aged 65-74, those aged 18-34 were more likely to have an intense fear of death and those aged 75+ were less likely to have an intense fear of death (Death Fear); people aged 18-34 were more likely to avoid thought of death when it entered mind (Death Avoidance); and those aged 18-34 and 35-49 were less likely to agree that death is an entrance to a place of ultimate satisfaction (Approach Acceptance). However, compared to younger and middle-aged adults, young-old adults were more likely to avoid thought of death when it entered mind and were less likely to agree that death is a natural aspect of life (Neutral Acceptance) if they belonged to the Moderate-ED class. These findings highlighted the resilience and death anxiety of older adults after experiencing multidimensional tornado impacts, which can provide important implications for the development of age-responsible mental health programs in post-disaster recovery.

